# Symptom Patterns of the Occurrence of Depression and Anxiety in a Japanese General Adult Population Sample: A Latent Class Analysis

**DOI:** 10.3389/fpsyt.2022.808918

**Published:** 2022-02-08

**Authors:** Huijie Lei, Chong Chen, Kosuke Hagiwara, Ichiro Kusumi, Hajime Tanabe, Takeshi Inoue, Shin Nakagawa

**Affiliations:** ^1^Division of Neuropsychiatry, Department of Neuroscience, Yamaguchi University Graduate School of Medicine, Ube, Japan; ^2^Department of Psychiatry, Hokkaido University Graduate School of Medicine, Sapporo, Japan; ^3^Faculty of Humanities and Social Sciences, Shizuoka University, Shizuoka, Japan; ^4^Department of Psychiatry, Tokyo Medical University, Tokyo, Japan

**Keywords:** comorbidity, depression, anxiety, symptom patterns, data-driven, latent class analysis

## Abstract

**Background:**

Given the high comorbidity and shared risk factors between depression and anxiety, whether they represent theoretically distinct disease entities or are just characteristics of a common negative affect dimension remains debated. Employing a data-driven and person-centered approach, the present study aims to identify meaningful and discrete symptom patterns of the occurrence of depression and anxiety.

**Methods:**

Using data from an adult sample from the Japanese general population (*n* = 403, including 184 females, age = 42.28 ± 11.87 years), we applied latent class analysis to identify distinct symptom patterns of depression (PHQ-9) and anxiety (STAI Y1). To empirically validate the derived class memberships, we tested the association between the derived classes and personal profiles including childhood experiences, life events, and personality traits.

**Results:**

The best-fitting solution had four distinct symptom patterns or classes. Whereas both Class 1 and 2 had high depression, Class 1 showed high anxiety due to high anxiety-present symptoms (e.g., “I feel nervous”) while Class 2 showed moderate anxiety due to few anxiety-absent symptoms (e.g., “I feel calm”). Class 3 manifested mild anxiety symptoms due to lacking responses on anxiety-absent items. Class 4 manifested the least depressive and anxiety-present symptoms as well as the most anxiety-absent symptoms. Importantly, whereas both Class 1 and 2 had higher childhood neglect and reduced reward responsiveness, etc. compared to Class 4 (i.e., the most healthy class), only Class 1 had greater negative affect and reported more negative life events.

**Conclusions:**

To our knowledge, this is the first latent class analysis that examined the symptom patterns of depression and anxiety in Asian subjects. The classes we identified have distinct features that confirm their unique patterns of symptom endorsement. Our findings may provide insights into the etiology of depression, anxiety, and their comorbidity.

## Introduction

Depressive and anxiety disorders (hereafter, depression, and anxiety) are the most common mental health diseases and causes enormous burden to the society (1). According to the Global Burden of Disease 2016 Study, both depression and anxiety ranked in the top 10 causes of Years Lived with Disability (YLD) in most countries and areas worldwide (2). Importantly, the two disorders often occur together, which causes greater distress (3), poorer treatment outcomes (4), and higher risk of suicide (5). For instance, data from the WHO World Mental Health Surveys indicated that 41.6% of people with 12-month major depressive disorder also had 12-month anxiety disorders (6).

In addition to the high comorbidity, the two disorders also have similar somatic symptoms, such as difficulty in sleeping and fatigue, and neuroendocrine profiles, such as the dysregulation of corticotropin-releasing factor (7). They also share substantial genetic risks (8) and have common psychopathological risk factors such as adverse childhood experience, negative life events, and enhanced sensitivity to punishment (9–12). Therefore, some researchers have argued that depression and anxiety are not exclusively distinct conditions, but are just different manifestations of a common negative affect dimension (13).

However, in spite of the high comorbidity and overlapping symptoms and risk factors, other researchers suggest that depression and anxiety are two separate entities, each with their unique features (14, 15). For instance, according to the Tripartite Model (16, 17), although depression and anxiety share a general distress or negative affect factor (e.g., feelings of upset, insomnia, restlessness, and irritability), depression is specifically associated with the absence of positive affect or anhedonia, while anxiety is specifically associated with physiological hyperarousal or somatic tension.

To resolve the dispute over the relation between depression and anxiety and provide insights into their psychopathology, recently researchers have started to employ latent class analysis (LCA) to identify the patterns of occurrence of depression and anxiety symptoms. Unlike traditional theory-driven and variable-centered approaches, LCA is a data-driven and person-centered technique that classifies individuals into more homogeneous groups (called classes) based on the patterns of symptoms by estimating the probabilities of symptom endorsement (18). This kind of symptom-level analysis may give a more explicit picture of the relationship between depression and anxiety symptoms (17).

By applying LCA to data of three national surveys (Australia, USA, and the Netherlands), Rhebergen et al. (19) identified three unique classes, one characterized by both high depression and anxiety symptoms (probability of symptom endorsement > 0.8), the second moderate depression and anxiety symptoms (> 0.4), and the third moderate depression symptoms only (with the endorsing probability of most anxiety symptoms being smaller than 0.1). In contrast, with another adult sample from the Dutch general population, general practices, and mental health organizations, the authors only identified two classes, one with both high depression and anxiety (probability of symptom endorsement > 0.6), the other high anxiety (>0.7) but moderate depression (>0.2). A class consists of individuals with predominantly high depression symptoms was not found in this Dutch sample. Hettema et al. (20) investigated an American sample of twins and identified four classes, one with almost no symptoms of depression and anxiety (<0.15) and the other three with low, moderate, or high symptoms of both depression and anxiety, respectively. These patterns remained stable at a follow-up survey conducted 1.5 years later. Curran et al. (21) applied LCA to a community-based sample of Irish older adults and identified four classes: one with no or low depression and anxiety (most symptoms < 0.15), the second high anxiety symptoms (most symptoms > 0.6), the third moderate depression (0.1–0.6) and high anxiety symptoms (>0.6), and the fourth both high depression and anxiety symptoms (most symptoms > 0.4). Curran et al. (21) was also the only study that applied LCA separately to males and females, which found that the above symptom patterns did not differ across gender. Taken together, in these previous studies, a subgroup of individuals with both high depression and anxiety has been consistently identified, while a subgroup with predominantly one symptom (depression or anxiety) has not been reliably identified. Although further empirical studies are still needed to clarify the mixed findings, these studies, from a data-driven and person-centered approach, have provided important insights that depression and anxiety tend to occur together in the same individuals.

To our knowledge, no research has explored the symptom patterns of depression and anxiety using LCA in Asian subjects. It has been reported that culture may shape the presentation of depression and anxiety symptoms (22). For instance, Asian individuals with depression and anxiety tend to overemphasize somatic symptoms, such as excessive fatigue and headache (23). They also tend to believe that negative emotions have cognitive and motivational utility and are less likely to engage in hedonic emotion regulation (24). Therefore, in the current study, we set out to investigate the symptom patterns of depression and anxiety in an adult sample from the Japanese general population using LCA. We then employed demographic data, psychological factors, and several established risk factors for depression and anxiety to validate the identified class memberships. Specifically, based on the Tripartite Model described above (16, 17), we used positive and negative affect and subjective wellbeing as psychological factors to validate the identified class memberships. We also used three well-known risk factors of depression and anxiety for the validation purpose, which include adverse childhood experiences, recent life events, and personality (9–12). The current study may provide important, novel insights into the association between depression and anxiety and the psychopathology of these disorders from a Japanese cultural perspective.

## Materials and Methods

### Subjects

The study was part of a larger study conducted between January and August 2014 that aimed to investigate the interaction of early life stress, recent life events, and vulnerability (e.g., personality) in affecting depression, anxiety, and wellbeing in the Japanese general adult population. All subjects were volunteers and were recruited by flyers posted on the campus of Hokkaido University (a major national university in Hokkaido Prefecture) and word of mouth. Questionnaires were distributed to the volunteers and returned anonymously. It took roughly 90 min to complete all the questionnaires. Of 853 volunteers, 455 participants (53.34%) gave written informed consent and responded to the questionnaires. Fifty-two participants were excluded due to incomplete responses on at least one item of the measures, resulting in a sample size of 403 (184 females, 45.7%; age = 42.28 ± 11.87 years, range 20–81 years). This study was approved by the ethics committees of Hokkaido University Hospital, Tokyo Medical University, and Yamaguchi University Hospital.

### Measurements

#### Depression and Anxiety

The 9-item Patient Health Questionnaire (PHQ-9) (25, 26) was employed to measure depressive symptoms in the past 2 weeks. The nine items represent the nine diagnostic criteria for major depression and were rated on a 4-point scale (0–3) which indicates the frequency of the symptom occurrence. The Cronbach's α of PHQ in the current study was 0.858. Anxiety symptoms were measured using the state anxiety subscale of State-Trait Anxiety Inventory Form Y (STAI-Y1) (27, 28). This subscale consists of 10 anxiety-present items (e.g., “I am tense,” “I feel nervous”) and 10 anxiety-absent items (e.g., “I feel calm,” “I feel satisfied”). Subjects answered how they feel at the particular moment in each statement on a 4-point scale, ranging from 1 (not at all) to 4 (very much so). Anxiety-absent items were reverse scored for calculating the total state anxiety score. The Cronbach's α of total STAI state anxiety, anxiety-present, and anxiety-absent subscales were 0.932, 0.870, and 0.948, respectively.

#### Demographic and Psychological Characteristics

Demographic information included age, gender, years of education, marital status, number of children, living status, employment, history of smoking, frequency of alcohol drinking (0 = none, 1 = sometimes, 2 = every day), comorbid physical diseases, history and family history of psychiatric diseases.

Furthermore, positive affect, negative affect, and subjective wellbeing were measured using the *Positive and Negative Affect Schedule* (PANAS) (29, 30) and *Subjective Well-being Inventory (SUBI)* (31, 32), respectively.

PANAS has 20 items and measures two broad domains of affect, positive and negative affect. Subjects described their feeling on positive and negative affect using a 6-point Likert scale, ranging from 1 (not at all) to 6 (extremely). Total scores for positive affect and negative affect subscales were separately calculated. The Cronbach's α of total PANAS, positive affect and negative affect subscales were 0.876, 872, and 0.898, respectively.

SUBI has 40 items and measures subjective wellbeing, including general wellbeing-positive affect, social support, and confidence in coping, etc., and subjective ill-being, including upsetability, physical ill-health, and deficiency in social contacts, etc. The wellbeing subscale consists of 19 items rated on a 3-point scale ranging from 1 (not apply to me) to 3 (apply to me). The ill-being subscale consists of 21 items rated on a 3-point scale (1 = always, 2 = sometimes, 3 = never). The total score for wellbeing and ill-being subscales were separately calculated. High scores indicate better states for both wellbeing and ill-being. The Cronbach's α of total SUBI, subjective wellbeing and subjective ill-being subscales were 0.912, 882, and 0.872, respectively.

#### Risk Factors

Several well-known risk factors of depression and anxiety, including childhood experiences, recent life events, and personality were measured using the Child Abuse and Trauma Scale (CATS) (33, 34), Life Experiences Survey (LES) (10, 35), and Behavioral Inhibition System and Behavioral Activation System Scales (BIS/BAS) (36, 37), respectively.

CATS has 38 items and measure childhood adverse experiences with three subscales: neglect/negative home environment, punishment and sexual abuse. Responses were based on a 5-point rating scale, where 0 indicates never and 4 indicates always. The mean score of the three subscales were used in current study. Cronbach's α of the three subscales was 0.859 (neglect), 0.504 (punishment), 0.824 (sexual abuse), respectively.

LES has 47 items and measures positive and negative life events happened in the past 6-month or 1 year and the impact of those events on a 7-point scale, ranging from−3 (extremely negative) to +3 (extremely positive). The total score of the two subscales were separately calculated.

BIS/BAS has 20 items and measures the sensitivity to cues of threat (i.e., BIS) and reward (i.e., BAS). BAS has 3 subscales: drive, fun-seeking, and reward responsiveness. Participants rated how they agreed with the statement of each item on a 4-point Likert scale, where 1 indicates strongly disagree and 4 indicates strongly agree. The total score for BIS and three subscales of BAS are used in the study. The Cronbach's α of BIS, drive, fun-seeking, and reward responsiveness were 0.785, 0.831, 0.729, and 0.736, respectively.

### Statistical Analysis

We conducted LCA in *Mplus* version 8.4 (38) using maximum likelihood estimation. Maximum likelihood is the default estimator in Mplus and has been commonly employed in previous studies. It has high statistical efficiency and has advantages in dealing with large numbers of items given small numbers of subjects (39). Based on the similarity displayed in subjects' response patterns, LCA categorizes them into more homogeneous groups, each with their own symptom endorsement probability. We used all the items of PHQ-9 and STAI-Y1 for the LCA. All items were dichotomized (i.e., absence of a symptom = 0, presence of a symptom = 1) due to a large number of items and a relatively small sample size. Specifically, following Holub et al. (40), the PHQ response option “not at all” was recoded into “absence of a symptom”, and options “several days,” “more than half the days,” and “nearly every day” were recoded into “presence of a symptom”. The state anxiety (STAI-Y1) response options “not at all” and “somewhat” were recoded into “absence of a symptom” while “moderately so” and “very much so” were recoded into “presence of a symptom”. Instead of LCA, we could have conducted a Latent Profile Analysis (LPA) using the original continuous items. However, based on simulation studies and expert recommendations, it has been suggested that at least 500 subjects are required for LPA [e.g., (41)] and each identified class should have at least 50–75 subjects [e.g., (42, 43)]. These requirements were not met in our dataset. Furthermore, given the small sample size, the frequency distributions of item responses were excessively skewed due to lack of response on the continuous response options (e.g., on 7 of the 9 items of PHQ, merely 1–14 subjects chose “more than half the days” or “nearly every day”). In cases like this, dichotomization of the responses and the employment of LCA are preferred (44). Nevertheless, we did run the LPA to see if we can reproduce the current LCA results. The LPA results are attached in the Supplementary Material. In brief, although with a small sample size in the identified classes, we could largely reproduce the current 4-class solution in LCA. The between-class differences in environmental and personality risk factors are also generally consistently between LPA and LCA.

To avoid convergence on solutions at a local maximum, we ran the LCA with 1,000 random starting values and 250 final stage optimizations. To select the best fitting model, we examined the Akaike information criteria (AIC), Bayesian information criteria (BIC), SSA-BIC (sample size adjusted BIC), entropy, the Lo-Mendell-Rubin adjusted likelihood ratio test (LMR-LRT), and the bootstrap likelihood ratio test (BLRT). Lower AIC, BIC, SSA-BIC indicate better fitting. LMR-LRT and BLRT with significant *p*-values indicate that the current k class model performs better than the previous k-1 class model. Entropy values bigger than 0.8 indicate good class separation. For model comparison, BIC and BLRT were prioritized, together with the interpretability of the derived latent classes (45, 46).

For the description of the winning model, the criteria of probability level per item were: low probability, ≤ 0.15; moderate probability, 0.16-0.59; high probability, ≥0.6 (47). After identifying the winning model, Chi-square test and Kruskal-Wallis test (because of the non-normality of the data according to the Kolmogorov–Smirnov test) conducted with IBM SPSS Statistics 26 were used to compare the demographic and psychological characteristics across the latent classes. Then multinomial logistic regression was performed to examine the relationship between risk factors and derived classes. One class was selected as the reference class in the logistic regression if its symptoms were close to healthy individuals. For the multinomial logistic regression, given the small sample size of the derived classes, environmental risk factors (i.e., CATS and LES) and personality were separately employed as independent factors. All non-binary variables were standardized to facilitate comparability. We did not detect any obvious multicollinearity issue (i.e., variance inflation factors all <5) with the independent variables of the logistic regression.

## Results

### LCA Analysis

The fitting results and number of subjects in each class are shown in Table 1. We chose the 4-class solution as the winning model because it performed better than that 3-class model based on the smaller BIC and significant LMR-LRT and BLRT. The 5-class solution performed no better than the 4-class solution because of the similar BIC values and non-significant *p*-value in LMR-LRT. The 4-class solution also had the highest interpretability and clinical relevance. The average latent class probabilities for the most likely latent class membership in the 4-class model were 0.980, 0.957, 0.933, and 0.970, respectively, indicating a high precision and reliability of the current model selection.

**Table 1:** Fitting statistics for latent class models from 2 to 5 classes.

**Number of classes**	**AIC**	**BIC**	**SSA-BIC**	**df**	**Entropy**	**LMR-LRT**	**BLRT**	**Number of subjects per class**				
								**N1**	**N2**	**N3**	**N4**	**N5**
2	14,071.406	14,319.340	14,122.607	62	0.941	*p* <0.000	*p* <0.000	220	183			
3	13,585.276	13,957.177	13,662.078	93	0.928	*p* <0.001	*p* <0.000	119	129	155		
**4**	**13,427.402**	**13,923.270**	**13,529.805**	**124**	**0.930**	***p*** **=** **0.041**	***p*** **<** **0.000**	**56**	**78**	**121**	**148**	
5	13,294.317	13,914.153	13,422.322	155	0.930	*p* = 0.448	*p* <0.000	25	69	77	92	140

*AIC, Akaike information criteria; BIC, Bayesian information criteria; SSA-BIC, sample size adjusted BIC; df, degrees of freedom; LMR-LRT, Lo-Mendell-Rubin adjusted likelihood ratio test; BLRT, bootstrap likelihood ratio test*.

*The results of the winning model are shown in bold*.

**Figure 1:**
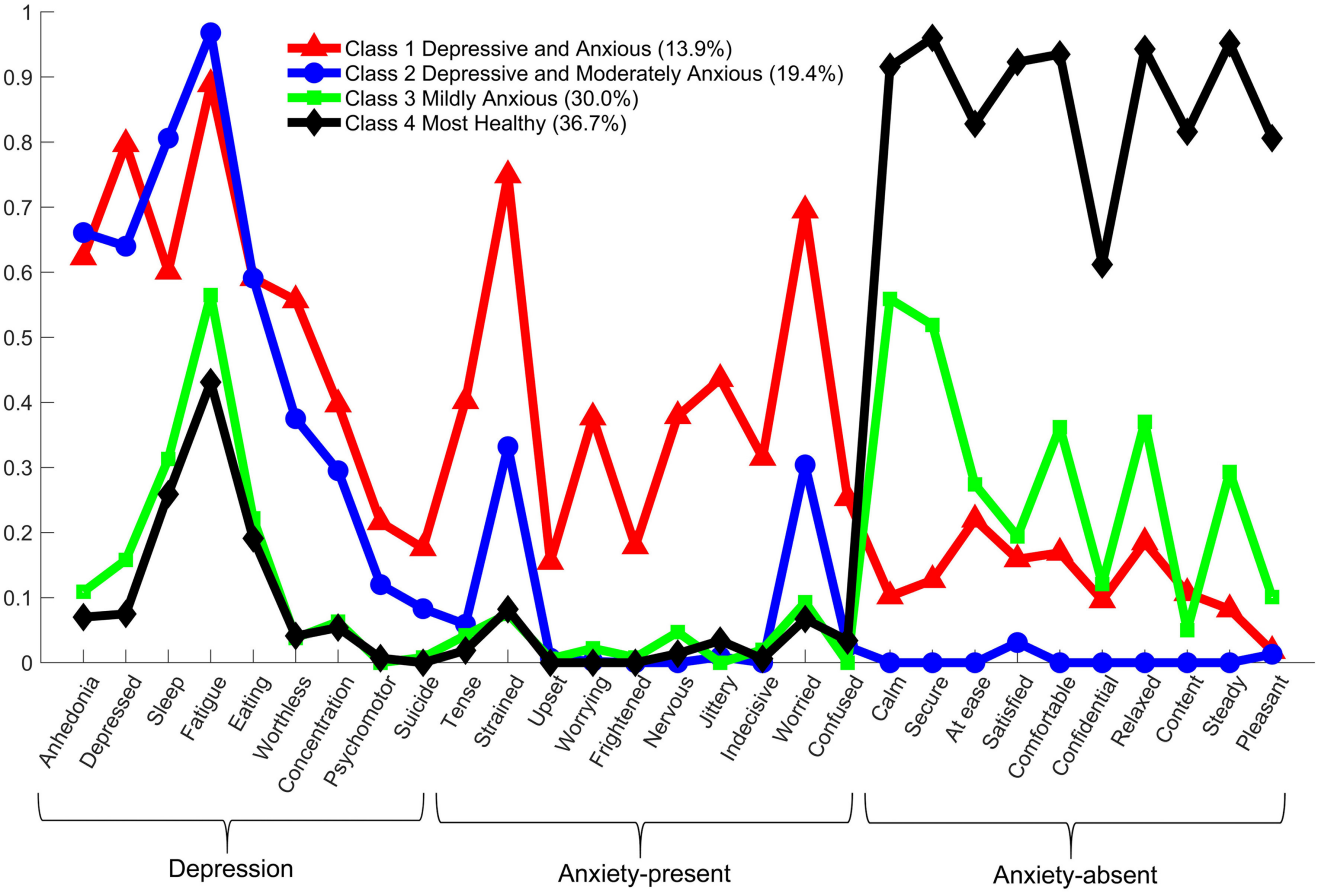
The probability of symptom endorsement by each latent class (i.e., conditional probability).

The probabilities of symptom endorsement for each class of the winning model are presented in Figure 1. Class 1 (*n* = 56, 13.9% of subjects) was characterized by moderate to high probabilities of most depression and anxiety-present symptoms (0.15–0.90) and thus labeled “Depressive and Anxious”. The second class (*n* = 78, 19.4%) was characterized by moderate to high probabilities of most depressive symptoms (0.25–1.0), moderate probabilities of two anxiety-present symptoms (i.e., strained and worried, 0.3–0.4), and the lowest probabilities of anxiety-absent symptoms (e.g., calm, secure, < 0.1). This class was thus labeled “Depressive and Moderately Anxious”. Class 3 (*n* = 121, 30.0%) and Class 4 (*n* = 148, 36.7%) were characterized by moderate probabilities (0.2–0.6) of somatic symptoms (sleep disturbances, feeling tired and trouble in eating), while Class 4 was notably different from Class 3 for its high probabilities of endorsing anxiety-absent symptoms (>0.6). Therefore, Class 3 and 4 were labeled “Mildly Anxious” and “Most Healthy”, respectively.

### Demographic and Psychological Characteristics

Table 2 displays demographic and psychological characteristics across the four derived classes. There was no significant difference among the four classes in demographic variables, including age, gender distribution, years of education, marital status, number of children, living status, employment, smoking history, frequency of alcohol drinking, the presence of comorbid physical disease, history and family history of psychiatric disease.

**Table 2:** Demographic and psychological characteristics of the 4 latent classes.

	**Class 1 depressive and anxious (*n* = 56)**	**Class 2 depressive and moderately anxious (*n* = 78)**	**Class 3 mildly anxious (*n* = 121)**	**Class 4 most healthy (*n* = 148)**
**Demographic**				
Age (years)	39.52 (11.319)	42.17 (10.777)	43.00 (11.763)	42.79 (12.651)
Gender: male	53.85%	47.96%	53.24%	52.47%
Education years	15.38 (1.996)	14.54 (2.383)	14.98 (2.280)	14.89 (2.164)
Marital status: unmarried	32.69%	28.57%	18.84%	21.25%
Number of children (0 or ≥1)	60.78%	59.79%	67.63%	70.63%
Living alone	14.00%	16.84%	25.90%	19.62%
Employment: homemakers	7.69%	6.32%	12.41%	14.47%
Smoking history	48.08%	51.02%	47.48%	49.38%
Frequency of alcohol drinking	1.13 (0.634)	1.10 (0.616)	1.15 (0.667)	1.10 (0.636)
Comorbidity of physical diseases	26.92%	24.74%	16.79%	20.00%
History of psychiatric diseases	9.62%	4.08%	2.88%	5.56%
Family history of psychiatric diseases	9.62%	16.49%	12.41%	8.64%
**Psychological characteristics**				
PHQ-9 total score	7.679 (6.043)^a^	6.051 (3.864)^**a**^	1.752 (1.823)^b^	1.318 (1.462)^b^
State anxiety total score	54.429 (8.549)^a^	48.026 (3.971)^a^	40.686 (4.227)^b^	30.318 (5.682)^c^
Anxiety-present total score	21.982 (5.594)^a^	15.218 (2.836)^b^	13.140 (2.599)^c^	12.351 (2.539)^c^
Anxiety-absent total score	17.554 (4.914)^a^	17.192 (2.594)^a^	22.455 (2.918)^b^	32.034 (4.293)^c^
PANAS positive affect	30.821 (8.415)	29.333 (6.664)^a^	30.992 (7.155)	33.500 (7.730)^b^
PANAS negative affect	31.661 (7.643)^a^	26.500 (6.920)^b^	23.802 (7.107)^b^	20.493 (7.111)^c^
Subjective wellbeing	36.125 (6.655)^a^	34.962 (5.854)^a, b^	38.264 (5.321)^a, c^	42.777 (5.519)^d^
Subjective ill-being	45.411 (6.347)^a^	49.321 (5.575)^a^	53.554 (4.950)^b^	55.142 (5.049)^b^

*Different superscript indicates significant difference at p < 0.05, Bonferroni corrected*.

As shown in Table 2, consistent with the class definitions, Class 1 and 2 had significantly higher PHQ and state anxiety total scores than Class 3 and 4. Importantly, whereas Class 1 and 2 did not differ from each other in terms of PHQ-9 and state anxiety total scores (*p* > 0.05), Class 1 had significantly higher anxiety-present score than Class 2 (*p* < 0.05). Furthermore, Class 1 and 2 also had similar subjective wellbeing and ill-being scores, while Class 1 had more enhanced negative affect than Class 2 (*p* < 0.05). Only Class 2 had significantly lower positive affect than Class 4 (*p* < 0.05), which is consistent with its lowest probabilities of endorsing anxiety-absent symptoms.

Meanwhile, Class 3 and 4 had similar PHQ score, while Class 3 had significantly higher anxiety than Class 4 due to less anxiety-absent (*p* < 0.05) but not more anxiety-present symptoms. Class 3 also had greater negative affect and lower subjective wellbeing than Class 4 (both *p* < 0.05).

### Risk Factors

To further validate the identified class memberships, we next used commonly studied environmental and personality risk factors to predict the class memberships with multinomial logistic regression. For this purpose, Class 4 (Most Healthy) was selected as the reference class because it was the closest to healthy individuals.

The results of the multinomial logistic regression with environmental risk factors are shown in Table 3. Compared to the reference class, whereas both Class 1 and 2 had higher childhood neglect or a more negative home environment (OR = 1.709 and 1.556, respectively, both *p* < 0.01), only Class 1 reported more negative life events in the past 6-month or 1 year (OR = 1.536, *p* < 0.01).

**Table 3:** Environmental risk factors predicting class membership: odds ratios and 95% confidence intervals from multinomial logistic regression.

	**Class 1 depressive and anxious (*n* = 56)**	**Class 2 depressive and moderately anxious (*n* = 78)**	**Class 3 mildly anxious (*n* = 121)**
CATS neglect	**1.709 (1.202–2.429)** ^**^	**1.556 (1.119–2.162)** ^**^	1.177 (0.855–1.620)
CATS punishment	1.195 (0.841–1.697)	0.948 (0.696–1.291)	0.968 (0.737–1.271)
CATS sexual abuse	0.862 (0.636–1.169)	0.930 (0.709–1.219)	0.903 (0.661–1.234)
LES positive life events	0.695 (0.478–1.011)	0.790 (0.588–1.061)	0.848 (0.663–1.084)
LES negative life events	**1.536 (1.142–2.066)** ^**^	1.281 (0.953–1.721)	0.971 (0.702–1.342)

*Class 4 served as the reference class*.

** *p < 0.01*.

*CATS, Child Abuse and Trauma Scale; LES, Life Experiences Survey*.

*Statistically significant results are shown in bold*.

The results of the multinomial logistic regression with personality risk factors are shown in Table 4. Compared to the reference class, all three classes reported significantly higher BIS and lower BAS reward responsiveness, with the change in Class 1 and 2 being greater than that in Class 3 (for BIS, OR = 3.975, 3.480, and 1.416, respectively; for reward responsiveness, OR = 0.389, 0.437, 0.588, respectively). Class 1 and 2 also had higher BAS fun-seeking (OR = 1.714 and 1.797, respectively). Thus, while Class 1 and 2 had higher childhood neglect and BIS and lower reward responsiveness, only Class 1 had more negative life events.

**Table 4:** Personality risk factors predicting class membership: odds ratios and 95% confidence intervals from multinomial logistic regression.

	**Class 1 depressive and anxious (*n* = 56)**	**Class 2 depressive and moderately anxious (*n* = 78)**	**Class 3 mildly anxious (*n* = 121)**
BIS	**3.975 (2.585–6.111)** ^***^	**3.480 (2.376–5.098)** ^***^	**1.416 (1.070–1.873)** ^*^
BAS Drive	0.852 (0.516–1.408)	0.692 (0.443–1.082)	1.099 (0.771–1.566)
BAS Fun-seeking	**1.714 (1.095–2.684)** ^*^	**1.797 (1.193–2.706)** ^**^	0.903 (0.636–1.282)
BAS Reward responsiveness	**0.389 (0.232–0.651)** ^***^	**0.437 (0.277–0.691)** ^***^	**0.588 (0.405–0.854)** ^**^

*Class 4 served as the reference class*.

* *p < 0.05*,

** *p < 0.01*,

*** *p < 0.001*.

*BIS, Behavioral Inhibition System; BAS, Behavioral Activation System*.

*Statistically significant results are shown in bold*.

## Discussion

In the present study, we investigated the symptom patterns of the occurrence of depression and anxiety by applying LCA in a sample of Japanese general population. We found that a 4-class solution best described our data. Specifically, Class 4 (Most Healthy) manifested the least depressive and anxiety-present symptoms as well as the most anxiety-absent symptoms; Class 3 (Mildly Anxious) manifested mild anxiety symptoms due to lacking responses on anxiety-absent items; Class 1 (Depressive and Anxious) had both high depressive and anxiety symptoms; Class 2 (Depressive and Moderately Anxious) displayed predominantly depression and moderate anxiety symptoms, and the moderate anxiety symptoms were the result of lacking response on anxiety-absent symptoms. Consistent with these unique patterns of symptom occurrence, we found that each class had distinct psychological characteristics and was associated with different risk factors.

To our knowledge, this is the first LCA study that examined the symptom patterns of depression and anxiety in a sample of Japanese general population. In line with findings from Western studies (19–21), we identified one class of subjects with both high depressive and anxious symptoms. However, we did not identify a predominantly high anxiety class (19, 21). That is, high anxiety tends to co-occur with high depression rather than occur alone. In this regard, this finding is somewhat inconsistent with the Tripartite Model which argues that depression and anxiety are different entities (16, 17). Furthermore, whereas Western studies have generally identified one class with almost no symptoms of depression and anxiety (20, 21), here even the healthiest class (Class 4) in our study showed moderate probability of endorsing symptoms in terms of sleep and eating problems and fatigue. This is perhaps explained by the observation that Eastern individuals tend to emphasize somatic symptoms (23). Among subjects with high depressive symptoms (Class 1 and 2), one-third (Class 1) also showed high anxiety symptoms, which is generally consistent with previous reports that among individuals with depression, roughly half suffer from anxiety (6). Notably, we did not identify a depression only class and the explanation of such result, we believe, has its cultural roots. In Asia, the common value of “conformity to norms, emotional self-control, collectivism, family recognition through achievement” [(48), p. 941] is strong and any deviation from the common value is considered inappropriate. As a result, people are afraid of being labeled “weak character” and while reporting depressed mood, they tend to in the meantime emphasize somatic and anxiety symptoms (23).

Another novelty of the current study was that we distinguished anxiety-present (“I feel nervous”) and anxiety-absent (e.g., “I feel calm”) items for the LCA. Although this distinction is common in anxiety-related inventories including the current STAI, few studies have included this distinction in LCA research of depression and anxiety. Intriguingly, we found that in two classes with high depression, one was accompanied by high anxiety-present symptoms (Class 1) while the other few anxiety-absent symptoms (Class 2). That is, in terms of anxiety, Class 1 was characterized by high negative symptoms while Class 2 low positive symptoms. This is consistent with their common and unique associations with other psychological characteristic and risk factors (in comparison to the reference class). Specifically, whereas both classes had higher childhood neglect (or a more negative home environment), behavioral inhibition, and fun-seeking, as well as reduced reward responsiveness, only Class 1 had greater negative affect and experienced more negative life events in the past 6 months or 1 year. Furthermore, only Class 2 had lower positive affect than Class 4. Previous studies have identified adverse childhood experiences in particular neglect and negative life events as risk factors of depression and anxiety (9, 10). Here, we confirmed these findings and further showed that they are only partially correct: whereas both Class 1 and 2 were associated with greater childhood neglect (or a more negative home environment), only Class 1 reported more negative life events in the past 6 months or 1 year. Therefore, negative life events are perhaps more sensitive risk factors for negative symptoms of anxiety rather than depression or anxiety due to lack of positive symptoms. Both Class 1 and 2 were associated with enhanced fun-seeking, which may reflect that fact that individuals with high levels of depression or moderate to high levels of anxiety tend to seek out novel, rewarding experiences in order to feel better. The etiological and psychopathological risk factors of this symptom pattern remain to be investigated by future studies.

Classes 1-3 were characterized by higher BIS and lower reward responsiveness on the BAS, with the change in the first two classes being greater. This is consistent with the notion that enhanced sensitivity to punishment and reduced sensitivity to reward are characteristics of depression, anxiety, and negative affect in general (11, 12, 49, 50).

We should also consider several limitations of the study when interpreting our findings. Firstly, we might have lost some information and overestimated the endorsement of symptoms by dichotomizing the responses of the depression and anxiety scales, and future studies with larger sample sizes are required to confirm our findings with continuous variables using LPA. Secondly, our subjects were primarily recruited on campus by flyers and word of mouth in Hokkaido, one of 47 prefectures in Japan. Caution, therefore, should be taken when generalize our findings to the whole Japanese general population. Thirdly, given our sample size, we were unable to apply LCA to males and females separately. It will be interesting for future research to investigate if the symptom patterns we identified exist in both genders. Fourthly, all scales in the current study were self-report measures, childhood experience and recent life events were also self-reported and assessed retrospectively. Our results, therefore, may suffer from recall and expectation bias. Future research should employ objective measures to confirm our findings. Furthermore, the punishment subscale of CATS had a somewhat low internal consistency as indicated by the Cronbach's α (i.e., 0.504). Further research may be needed to confirm the reliability of the scale in Japanese subjects. Nevertheless, the Cronbach's α of the punishment subscale was close to that reported in Japanese subjects in previous studies [(51), in which Cronbach's α = 0.58; (52), in which Cronbach's α = 0.55] as well as subjects from UK [(53), Cronbach's α = 0.63] and Iran [(54), Cronbach's α = 0.63]. Our results are also consistent with previous studies in that the Cronbach's α of the punishment subscale was lower than the other two subscales of CATS [i.e., neglect and sexual abuse, (51–54)].

The current study was cross-sectional and future longitudinal studies are required to verify whether our identified symptom patterns are stable over time. Furthermore, to better uncover the underlying mechanism of depression and anxiety, multiple data such as those of neural circuitry, genetic, and molecular may be included in future LCA studies (55, 56). Lastly, the current study used a non-clinical sample, future study should verify our results in clinical patients in order to shed light on the pathophysiology of depression and anxiety.

In conclusion, the current study identified four distinct symptom patterns of the occurrence of depression and anxiety in a sample of Japanese general population. We found two classes with high depression, with one showing concurrent high anxiety. Both classes have distinct features that confirm their unique patterns of symptom endorsement. Our findings may provide insights into the etiology of depression, anxiety, and their comorbidity, and have implications for dissecting the heterogeneity and individualizing the treatment of these disorders.

## Data Availability Statement

The data that support the findings of this study are available from the corresponding author upon reasonable request.

## Ethics Statement

The studies involving human participants were reviewed and approved by the Ethics Committees of Hokkaido University Hospital, Tokyo Medical University, and Yamaguchi University Hospital. The patients/participants provided their written informed consent to participate in this study.

## Author Contributions

CC, HT, TI, and SN: conceptualization and design. IK and TI: investigation. HL, CC, and KH: data analysis. HL and CC: manuscript preparation. All authors revised the manuscript. All authors contributed to the article and approved the submitted version.

## Conflict of Interest

Author IK has received honoraria from Daiichi Sankyo, Dainippon Sumitomo Pharma, Eisai, Eli Lilly, Janssen Pharmaceutical, Lundbeck, Meiji Seika Pharma, Mochida Pharmaceutical, MSD, Mylan, Novartis Pharma, Ono Pharmaceutical, Otsuka Pharmaceutical, Pfizer, Shionogi, Shire, Taisho Toyama Pharmaceutical, Takeda Pharmaceutical, Tsumura, and Yoshitomiyakuhin, and has received research/grant support from Asahi Kasei Pharma, Astellas, Daiichi Sankyo, Dainippon Sumitomo Pharma, Eisai, Eli Lilly, Mochida Pharmaceutical, Novartis Pharma, Otsuka Pharmaceutical, Pfizer, Shionogi, Takeda Pharmaceutical and Tanabe Mitsubishi Pharma. Author TI has received personal fees from Mochida Pharmaceutical, Takeda Pharmaceutical, Eli Lilly, Janssen Pharmaceutical, MSD, Taisho Toyama Pharmaceutical, Yoshitomiyakuhin, and Daiichi Sankyo; grants from Shionogi, Astellas, Tsumura, and Eisai; and grants and personal fees from Otsuka Pharmaceutical, Dainippon Sumitomo Pharma, Mitsubishi Tanabe Pharma, Kyowa Pharmaceutical Industry, Pfizer, Novartis Pharma, and Meiji Seika Pharma; and is a member of the advisory boards of Pfizer, Novartis Pharma, and Mitsubishi Tanabe Pharma. Author SN has received personal fees from EA Pharma, Janssen Pharmaceutical, Kurasie, Kyowa Pharmaceutical Industry, Lundbeck, Meiji Seika Pharma, Mylan, MSD, Takeda Pharmaceutical, and Yoshitomiyakuhin; grants from Astellas, Daiichi Sankyo, Nihon Medi-physics, Novartis Pharma, Tanabe Mitsubishi Pharma, and grants and personal fees from Eisai, Eli Lilly, Dainippon Sumitomo Pharma, Mochida Pharmaceutical, MSD, Otsuka Pharmaceutical, Pfizer, Shionogi and Tsumura. The remaining authors declare that the research was conducted in the absence of any commercial or financial relationships that could be construed as a potential conflict of interest.

## Publisher's Note

All claims expressed in this article are solely those of the authors and do not necessarily represent those of their affiliated organizations, or those of the publisher, the editors and the reviewers. Any product that may be evaluated in this article, or claim that may be made by its manufacturer, is not guaranteed or endorsed by the publisher.
