# Supplementary material for: Symptom Patterns of the Occurrence of Depression and Anxiety in a Japanese General Adult Population Sample: A Latent Class Analysis
Source: Front Psychiatry. 2022 Feb 8;13:808918. doi: 10.3389/fpsyt.2022.808918 (PMC8861440; doi:10.3389/fpsyt.2022.808918)
Supplement: Supplementary file 1 [file Data_Sheet_1.pdf]

Symptom patterns of the occurrence of depression and anxiety in a Japanese general adult population sample: a latent class analysis

Supplementary material

The fitting statistics for LPA models with 2-4 classes are shown in **Table S1**. As can be seen, the first class of the 3-class solution and the 4-class solution had a sample size smaller than the typically recommended 50 (n=44 and 8, respectively). The symptom endorsement of each class for all 2-4 class solutions is shown in **Figure S1**.

The 2-class solution identifies a high depression and high anxiety class (similar to our Class 1 Depressive and Anxious in LCA) and a low depression and low anxiety class (similar to our Class 4 Most Healthy). Based on the literature review we provided in the Introduction of the manuscript, this 2-class solution is considered oversimplified.

The 3-class solution differs from the 2-class solution in that it further divides the high depression and high anxiety class in the 2-class solution into two separate classes: a high depression and high anxiety class (similar to our Class 1 Depressive and Anxious) and a mild depression and mild anxiety class (similar to our Class 3 Mildly anxious). The former class, however, has a sample size of only 44, smaller than 50 as suggested by previous studies discussed in the main text.

The 4-class solution further separates the high depression and high anxiety class in the 3-class solution to two classes: a high depression and high anxiety class (similar to our Class 1 Depressive and Anxious) and a moderate depression and moderate anxiety class (similar to our Class 2 Depressive and Moderately Anxious). The 4-class solution, therefore, is generally consistent with our results using LCA.

We further investigated the between-class differences with the 4-class solution using demographic and personal profiles, the results of which are reported in **Table S2-4**. As shown in **Table S2**, there are no between-class difference in any demographic variables except History of psychiatric diseases. The between-class differences in terms of PHQ and STAI total scores and Subjective wellbeing are generally consistent with our LCA results. As shown in **Tables S3** and **S4**, the between-class differences in terms of

environmental and personality risk factors are also generally consistent with our LCA results.

Table S1. Fitting statistics for 2-4 classes using LPA

Number of classes	AIC	BIC	SSA-BIC	df	Entropy	LMR-LRT	BLRT	Number of subjects per class			
								N1	N2	N3	N4
2	22199.645	22551.551	22272.318	88	0.945	p = 0.003	p < 0.000	186	217		
3	20806.860	21278.734	20904.308	118	0.960	p = 0.189	p < 0.000	44	154	205	
4	20034.154	20625.997	20156.377	148	0.966	p = 0.530	p < 0.000	8	33	158	204

Figure S1. Symptom endorsement for the 2-4 class solutions in LPA

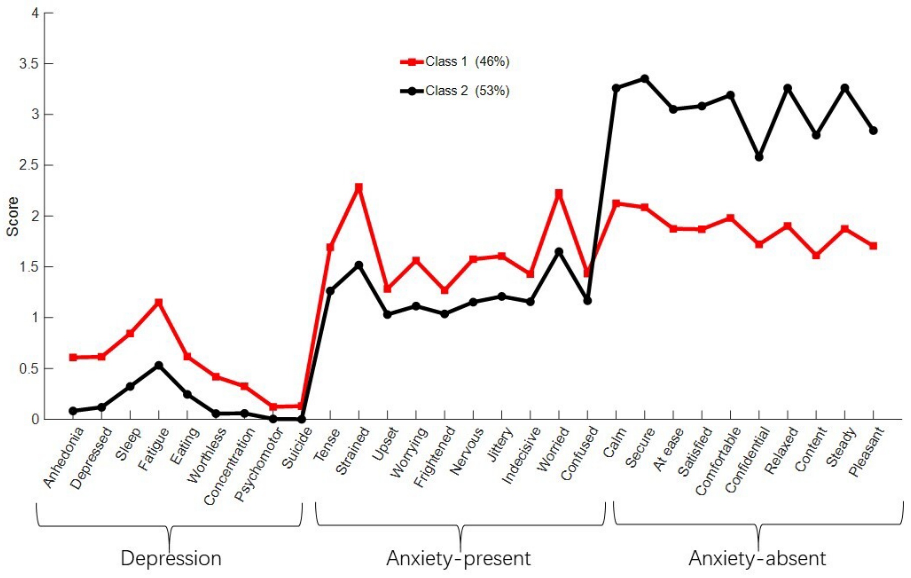

a), 2-class solution

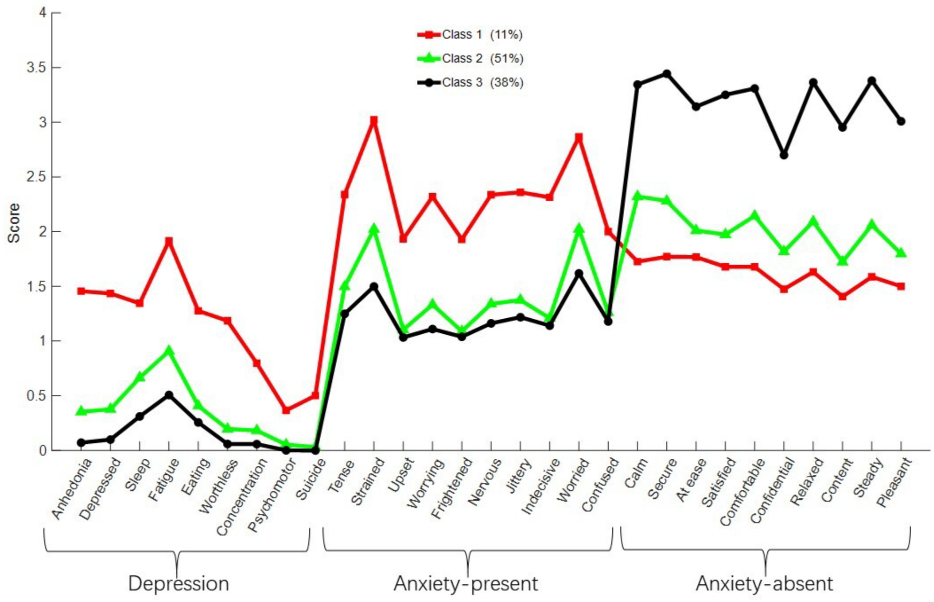

b), 3-class solution

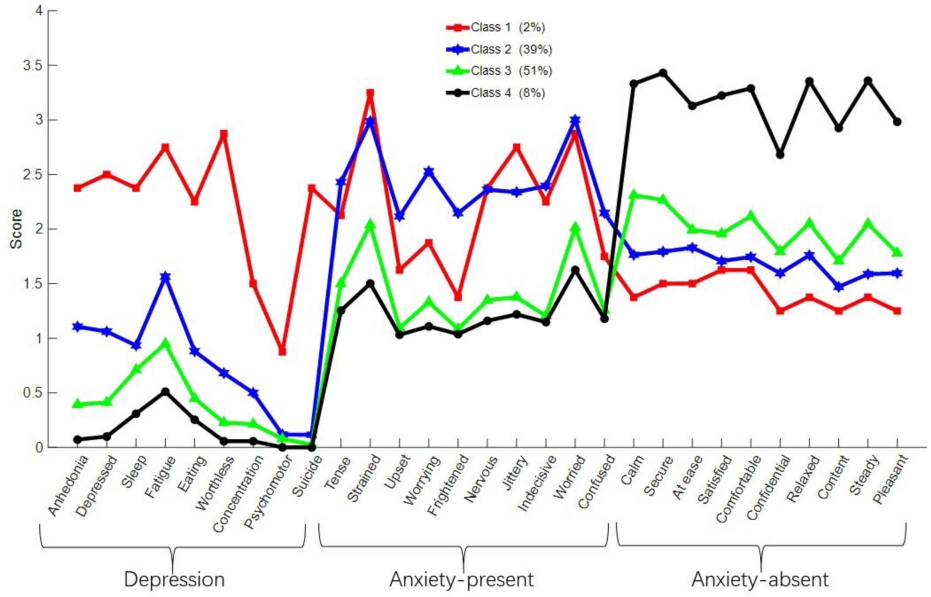

c), 4-class solution

Table S2. Demographic and psychological characteristics of the 4 latent classes in LPA

	Class 1 Depressive and Anxious (n=8)	Class 2 Depressive and Moderately Anxious (n=33)	Class 3 Mildly Anxious (n=204)	Class 4 Most Healthy (n=158)
**Demographic**				
Age (years)	36.88 (11.141)	41.91 (10.441)	42.14 (11.677)	42.81 (12.447)

Gender: male	37.50%	54.55%	54.43%	54.90%
Education years	14.38 (3.021)	15.12 (2.147)	14.82 (2.235)	15.03 (2.198)
Marital status: unmarried	37.50%	21.21%	18.35%	25.00%
Number of children (0 or ≥1)	50.00%	75.76%	69.62%	63.24%
Living alone	25.00%	12.12%	22.15%	20.10%
Employment: homemakers	12.50%	15.15%	13.92%	7.84%
Smoking history	62.50%	54.55%	48.10%	49.02%
Frequency of alcohol drinking	0.88 (0.641)	1.24 (0.614)	1.10 (0.643)	1.13 (0.640)
Comorbidity of physical diseases	37.50%	12.12%	20.89%	21.57%
History of psychiatric diseases	37.50% [a]	3.03% [b]	5.70% [b]	3.92% [b]
Family history of psychiatric diseases	12.50%	3.03%	10.76%	13.73%
**Psychological characteristics**				
PHQ-9 total score	19.875 (3.523) [a]	7.091 (3.924) [a,#]	3.422 (3.283) [b]	1.380 (1.558) [c]
State anxiety total score	58.125 (7.396) [a]	57.848 (7.076) [a]	44.211 (4.902) [b]	30.449 (5.474) [c]
Anxiety-present total score	22.250 (6.065) [a]	24.576 (4.373) [a]	14.275 (2.900) [b]	12.247 (2.475) [c]
Anxiety-absent total score	14.125 (4.941) [a]	16.727 (4.509) [a]	20.064 (3.418) [a]	31.797 (4.278) [b]
PANAS Positive affect	24.125 (7.954) [a]	31.515 (7.421) [a, b]	30.426 (7.276) [a]	33.430 (7.590) [b]
PANAS Negative affect	35.250 (9.543) [a]	32.061 (6.874) [a]	25.314 (7.249) [b]	20.563 (7.050) [c]
Subjective well-being	29.875 (6.749) [a]	37.000 (6.225) [a]	36.936 (5.850) [a]	42.506 (5.498) [b]
Subjective ill-being	34.750 (2.605) [a]	46.212 (5.122) [a]	51.608 (5.485) [b]	55.063 (5.077) [c]

Note: Different superscript indicates significant difference at p<0.05, Bonferroni corrected. [#]: p=0.089 compared to Class-1 after Bonferroni correction.

Table S3. Environmental risk factors predicting class membership in LPA: Odds ratios and 95% confidence intervals from multinomial logistic regression

	Class 1 Depressive and Anxious (n=8)	Class 2 Depressive and Moderately Anxious (n=33)	Class 3 Mildly Anxious (n=204)
CATS Neglect	**3.407 (1.691-6.862)** **	1.499 (0.992-2.266)†	1.206 (0.925-1.571)
CATS Punishment	1.089 (0.477-2.488)	1.048 (0.694-1.582)	1.063 (0.841-1.345)
CATS Sexual abuse	1.093 (0.711-1.681)	0.995 (0.634-1.561)	1.087 (0.806-1.467)
LES Positive life events	0.251 (0.038-1.658)	0.669 (0.417-1.072)	0.846 (0.685-1.046)
LES Negative life events	**2.279 (1.446-3.593)** ***	**1.436 (1.029-2.004)** *	1.041 (0.812-1.334)

Note: Class 4 served as the reference class. *p<0.05, **p<0.01, ***p<0.001, †p=0.055. CATS: Child Abuse and Trauma Scale; LES: Life Experiences Survey.

Table S4. Personality risk factors predicting class membership in LPA: Odds ratios and 95% confidence intervals from multinomial logistic regression

	Class 1 Depressive and Anxious (n=8)	Class 2 Depressive and Moderately Anxious (n=33)	Class 3 Mildly Anxious (n=204)
BIS	**8.828 (3.101-25.133)** ***	**3.484 (2.141-5.669)** ***	**1.892 (1.472-2.432)** ***
BAS Drive	0.369 (0.111-1.234)	0.754 (0.415-1.371)	1.033 (0.752-1.418)
BAS Fun-seeking	**2.921 (1.153-7.403)** *	**1.714 (1.022-2.872)** *	1.077 (0.795-1.458)
BAS Reward responsiveness	0.344 (0.16-1.016)†	**0.450 (0.248-0.815)** **	**0.551 (0.395-0.769)** ***

†p=0.053
